# Using the Columbia Suicide Severity Rating Scale to Assess Suicidality Among Young Women in the Urban Slums of Kampala Uganda: Baseline Findings from the TOPOWA Cohort Study

**DOI:** 10.3390/ijerph23020170

**Published:** 2026-01-29

**Authors:** Monica H. Swahn, Charles Natuhamya, Rachel Culbreth, Jane Palmier, Kate Mobley, Godfrey S. Bbosa, Gideon Matovu, Anna Kavuma, Paul Bukuluki, Godfrey Zari Rukundo, David Ndetei

**Affiliations:** 1 School of Public Health, Virginia Commonwealth University, Richmond, VA 23219, USA; 2 Uganda Youth Development Link, Kampala P.O. Box 12659, Uganda; 3 American College of Medical Toxicology, Phoenix, AZ 85028, USA; 4 School of Data Science and Analytics, College of Computing and Software Engineering, Kennesaw State University, Kennesaw, GA 30144, USA; 5 Department of Pharmacology & Therapeutics, Makerere University College of Health Sciences, Kampala P.O. Box 7072, Uganda; 6 Department of Social Work and Administration, School of Social Sciences, Makerere University, Kampala P.O. Box 7072, Uganda; 7 Department of Psychiatry, Mbarara University of Science and Technology, Mbarara P.O. Box 1410, Uganda; 8 African Center for Suicide Prevention and Research, Mbarara P.O. Box 379, Uganda; 9 Africa Institute of Mental and Brain Health, Nairobi P.O. Box 48423-00100, Kenya; 10 Department of Psychiatry, University of Nairobi, Nairobi P.O. Box 30197-00100, Kenya

**Keywords:** suicidality, suicide attempt, mental health, slums, suicidal ideation, poverty, young women, urban slums, Uganda, Columbia-Suicide Severity Rating Scale (C-SSRS), social determinants of health, prevention

## Abstract

The purpose of this study was to use the Columbia Suicide Severity Rating Scale (C-SSRS) to assess the prevalence and patterns of suicidality among young women living in poverty to guide effective, targeted interventions for vulnerable populations. Data were drawn from ‘The Onward Project On Wellbeing and Adversity’ (TOPOWA) study, a prospective cohort examining mental health in the context of social determinants of young women aged 18 to 24 years in Kampala’s urban slums. A cohort of 300 women, recruited from three study sites, participated in baseline assessments. Suicidality was assessed using the C-SSRS. Demographic and psychosocial factors and their associations with suicidality are presented. Of the 300 women participants, 66.0% had some secondary education and 62.0% had children, with most of them living with their children (81.7%). Suicidal thoughts were reported by 46.0%, and 17.3% had attempted suicide, with poisoning (23.1%) and hanging (21.2%) being the most common methods. The prevalence of suicidality in this population was very high, indicating significant unmet mental health needs. Since not all suicide attempts are associated with preceding thoughts or plans, it is crucial to consider a broader range of risk factors and warning signs. Social support systems and socioeconomic strengthening may be fruitful strategies for the prevention of suicidality in this population.

## 1. Introduction

Suicide is a critical public health concern globally, especially in Uganda, where poverty and legal context shape risk and response. Globally, suicide ranks as the fourth leading cause of death among adolescents and young adults aged 15–29 [1], and 77% of suicide deaths occur in low- and middle-income countries (LMICs) [2]. Additionally, the COVID-19 pandemic further exacerbated the structural and psychosocial risk factors for suicide [3]. In Uganda, suicide is a growing issue, with estimates indicating that 31% of youth in the slums experience suicidal ideation [4,5], which is significantly higher than the 16–22% reported among high-school youth in the United States [6].

The higher prevalence of suicidality in Uganda, particularly among those living in poverty, can be attributed to various socioeconomic and environmental factors that exacerbate mental health issues [5,7,8,9]. These factors include extreme poverty, limited access to education and health services, substance use, and exposure to violence, which are especially present in urban slums [10,11]. Poverty has consistently been linked to higher suicide risk [12,13]. Additionally, other socioeconomic factors at the community level have been linked to higher suicide risk, including community deprivation, family disagreements, land wrangles, unemployment, and lower education [13]. Addressing the social determinants of mental health is crucial for developing effective interventions to reduce the high rates of suicidal ideation and improve the overall well-being of youth in Uganda [7,8].

Youth living in slums or on the streets face even greater risks for suicidal behaviors and ideation, exacerbated by their challenging social, economic, and environmental conditions [4,12,14,15,16,17]. Slum environments are characterized by overcrowding, inadequate sanitation, and high levels of violence [10,14,18,19]. These environmental living conditions and exposures significantly impact mental health [20]. Family breakdowns, social isolation, substance use, and exposure to violence are prevalent and significantly impact the mental well-being of youth [21,22]. Youth living in the slums of Kampala therefore have a high prevalence of suicidal ideation, ranging from 23.5% to 30.6% [5,10]. These findings are comparable to recent research on youth in Kenya, a neighboring country in East Africa, which reported the prevalence of suicidal thoughts (26.8%), plans (14.9%), and attempts (15.7%) [23].

Furthermore, in Uganda, suicidality is also a significant public health concern among populations affected by HIV/AIDS, psychological distress, and post-conflict environments [24,25]. Research highlights the varying prevalence of suicidality in different contexts within Uganda. Studies among people living with HIV in Uganda have documented elevated prevalence of suicidal ideation and attempts across both urban and semi-urban clinical settings [24,26]. It is estimated that 3.7% of 15–24-year-olds in Uganda are HIV-positive [27], and that the prevalence of HIV in the slums is considerably higher [28]. For this reason, understanding the link between HIV and suicidality is important for contextualizing the high prevalence of suicidal thoughts and behaviors among youth living in the slums of Kampala. Negative life experiences, including child maltreatment and humanitarian crises, are significant drivers of psychological distress and suicidal ideation [18,29]. Studies in post-conflict Northern Uganda have shown high rates of post-traumatic stress disorder (11.8%) [21] and suicidal ideation (8.6%) and attempts (2.3%) [30]. The COVID-19 pandemic was also associated with marked increases in depression in Uganda [31]. The living conditions in slums are often characterized by overcrowding, lack of clean water, electricity, and sanitation, and high exposure to crime and violence, severely impact mental health [32]. The relationship between the living conditions in the slums and poor mental health may be explained by the link between place and mental health [20]. Additionally, social support networks, which are an important contributor to good mental health, are often weak or absent in the slums due to the high mobility and instability present in slum communities. Social support networks in the slums are adversely impacted by slum clearance or forced evictions, which are common in African cities. A result of slum clearance and forced evictions is that slum residents rarely live in the same place for more than a few years, further weakening social support networks [33,34]. Conditions in the slums, particularly social isolation, alcohol and substance use, and exposure to violence and abuse, significantly impact the mental well-being of youth in the slums [10,14].

Overall, the prevalence of suicidality in Uganda varies across different populations and settings, with rates influenced by factors such as HIV/AIDS, psychological distress, post-conflict environments, and crises like the COVID-19 pandemic [21,24,26,30,35,36]. These findings underscore the importance of the individual and social context and the need for targeted interventions and mental health support to address the complex interplay of factors contributing to suicidality in Uganda. However, to date, these types of interventions are lacking. Suicide prevention research also remains relatively scarce in Uganda, which is highly problematic given the scope of suicidality. In fact, at the time of preparing this article, we could not find any research findings from any prospective cohort studies designed to measure suicidality in Uganda, nor could we find any studies using the Columbia Suicide Severity Rating Scale (C-SSRS) [37], which is currently considered the golden standard for both suicide research and clinical assessments by many. The C-SSRS has been validated through extensive research and is considered reliable in different populations and settings [37,38,39,40] and is widely used and endorsed for research and clinical assessments [39,40,41].

This article presents findings from the baseline assessment of the prospective TOPOWA cohort study. The TOPOWA study aims to examine the longitudinal mechanistic pathways of suicidality among young women aged 18 to 24 living in three slums across Kampala. The broader study will evaluate whether socioeconomic empowerment and skills-based training may mitigate adverse social determinants of mental health [7,42,43]. This work is guided by a social determinant of mental health framework emphasizing economic hardship, access to services and social support [7,8]. In Kampala’s slums, economic hardship, unemployment, and limited educational opportunities contribute to psychological distress and increased suicidality [4,43,44,45]. Access to health services, particularly mental healthcare, remains limited in Uganda’s slums, compounding untreated mental health conditions [31,46,47].

This paper contributes to the epidemiologic evidence of suicidal behavior among young women in Kampala to inform prevention and intervention planning. We address the following: (1) the prevalence of suicidal thoughts, plans, and attempts; (2) the methods used in suicide attempts; (3) the patterns of suicidal thoughts and plans to suicide attempts; and (4) key sociodemographic characteristics associated with suicidality among young women living in urban poverty in Kampala. We used the C-SSRS to estimate lifetime and past-month suicidal thoughts and behaviors, enabling cross-national comparisons.

## 2. Methods

### 2.1. Recruitment

Between July 2023 and November 2023, 300 young women aged 18 to 24 years were recruited for a prospective observational cohort study conducted at three sites in Kampala—Bbanda, Bwaise, and Makindye—as part of TOPOWA study. The study’s goal is to investigate the mechanistic pathways leading to mental illness among this population and the methods have been described previously [48,49,50,51]. The present analysis is based specifically on the baseline data. Participants were eligible for study enrollment if they identified as female, were between the ages of 18 and 24 years, resided within 2 km of a Uganda Youth Development Link (UYDEL) vocational training center, and had completed at least a primary five education level. Women were excluded from participation if they were pregnant, had a significant intellectual disability, or were suffering from severe mental illness or substance use disorders that required hospitalization. These criteria reflect the community-based, non-clinical design of the cohort.

Out of 495 women screened for eligibility, 137 did not meet the study criteria, and 58 did not attend the enrollment session, resulting in an 83.8% participation rate among those eligible. A total of 150 participants were allocated to each of the two cohort groups: one intervention group receiving vocational training and psychosocial support, and a community comparison group. Both groups were matched on factors such as education level, average monthly income, and number of children. Although the TOPOWA cohort will include 10 assessments over the course of 27 months (roughly every 3 months), only baseline data were used in this assessment. Therefore, both groups were pooled for analysis.

At baseline, participants completed a structured survey administered by research assistants, which captured a broad array of information related to demographics, psychosocial factors, and life experiences, alongside other study-specific measures. The study adhered to the ethical principles outlined in the Declaration of Helsinki. The research was reviewed and approved by the Kennesaw State University Institutional Review Board, the Makerere University School of Health Sciences Research and Ethics Committee (MAKSHSREC-2023-532), and the Uganda National Council for Science and Technology (UNCST) under protocol HS2959ES. Written informed consent was obtained from each participant before their inclusion in the study. Participants received compensation, consistent with local IRB guidelines, for completing the survey and participating in other elements of the data collection process. This study did not require a clinical trial registration.

### 2.2. Measures

#### 2.2.1. The Columbia Suicide Severity Rating Scale (C-SSRS)

The C-SSRS, designed to quantify the severity of suicidal ideation and behavior, was used in this study [37]. The psychometric properties of the C-SSRS have been evaluated and conclusions indicate that the measure has good internal consistency, and that the total score and the most severe ideation item were correlated with other measures of suicidality [38]. While the authors were unable to find any publications using the C-SSRS in Uganda, it has been used in Tanzania and South Africa, and recently in Kenya [23,52,53]. In this community-based study, the C-SSRS was used as a structured assessment of suicidal ideation and behavior, rather than as a diagnostic instrument.

The C-SSRS assessed suicidal behaviors across two time periods: lifetime and the past month. The lifetime assessment was used because prior suicidal thoughts and behaviors—regardless of timing—are consistently identified in the literature as among the strongest indicators of future suicide risk and ongoing vulnerability [54,55].

Suicidal thought was assessed using the first 4 questions of the C-SSRS which measured a wish to be dead, nonspecific active suicidal thoughts, suicidal thoughts with methods, and suicidal intent. A woman was classified as having a suicidal thought when she affirmed responses to any of the 4 questions.

Suicidal attempts were classified into full attempts, interrupted attempts, and aborted (self-interrupted) attempts. To assess the history of actual attempt, women were asked; “Have you made a suicide attempt at any point in your lifetime?” To determine the history of interrupted attempts, women were asked; “Has there been a time in your life when you started to do something to end your life but someone or something stopped you before you actually did anything?” To assess the history of aborted (self-interrupted) attempts, women were asked; “In your lifetime, has there been a time when you started to do something to try to end your life, but you stopped yourself before you actually did anything?” Participants were first presented with the actual suicide attempt question, followed by the interrupted and aborted suicide attempt ones. Each suicide attempt subtype was dichotomized as the presence (1) or absence (0) of a history of that particular subtype of a suicide attempt.

Classifications of suicide attempts followed previously outlined strategies in a similar study [56]. Individuals were classified in the actual suicide attempt group if they endorsed an actual suicide attempt, regardless of whether they reported any interrupted or aborted attempts (i.e., if an individual reported both an actual suicide attempt and an interrupted or aborted attempt, they were coded to be in the actual suicide attempt group). Individuals were classified in the interrupted/aborted suicide attempt group if they endorsed either an interrupted suicide attempt, aborted suicide attempt, or both without endorsing an actual suicide attempt. Individuals were classified in the non-attempt group if they denied a history of any suicide attempt and in the attempter group, otherwise. Suicidal methods were measured by asking; “What are the methods of suicide that you have tried?” Multiple responses were allowed for this question.

#### 2.2.2. Demographic Survey Measures

A demographic survey questionnaire was used to study the socioeconomic and family characteristics of the study population. Information collected included age, education level, parental survival status, parenting status (including ages and number of children), and socioeconomic characteristics including employment status, income, and food insecurity. The wealth quintile measure was derived from information on household assets and characteristics (e.g., television, bicycles, cows, and poultry). Factor analysis was applied to construct the wealth quantiles.

#### 2.2.3. Depression Measure

The Patient Health Questionnaire (PHQ-9) [57] was used to measure depressive symptoms, which we will refer to herein as depression for simplicity. The participants were asked how often in the past 2 weeks they had been bothered by nine problems, including “feeling down, depressed, or hopeless” and “poor appetite or overeating”. Responses were coded ranging from 0 (not at all) to 3 (nearly every day). The PHQ-9 sum score ranges from 0 to 27, and a higher score indicates more severe depressive symptoms. In this study, a sum score of 8 or greater suggested significant depression. The scale’s internal consistency was high (Cronbach’s α = 0.79).

#### 2.2.4. Intimate Partner Violence (IPV) Measure

The 13-item WHO violence against women tool [58] was used to assess IPV. The scale has three constructs: psychological, physical, and sexual violence. Respondents were asked whether their current husband/partner, or any other partner, ever insulted or made them feel bad about themselves, physically forced them to have sexual intercourse when they did not want to, etc. Responses were “yes” or “no”. A woman was categorized to have ever experienced IPV if they agreed to any of the questions.

#### 2.2.5. Quality of Life (QoL) Measure

The Ugandan Youth Quality of Life index [59,60] was used to measure QoL. The QoL tool originally included 36 questions, divided into a two-part scale: 18 questions rated for satisfaction and 18 other questions rated for importance. During analysis, 17 items were considered, excluding 1 item that was dropped during the psychometric analysis. The tool has 3 subscales: living conditions and lifestyle (7 items), social relationships (5 items), and personal independence (5 items), with all items ranging from 1 (very dissatisfied) to 5 (very satisfied) in the satisfaction part and 1 (not at all important) to 5 (indispensable) in the importance part. The sum score was computed for the single-item measure. The satisfaction scores were initially weighted at the item level, producing a possible range of 0 to 20. The weighted score was rescaled to a 100-point scale ranging from 0 (poor QoL or poor well-being) to 100 (perfect QoL or well-being) to ease interpretation.

### 2.3. Statistical Analysis

Descriptive statistics, including frequency distributions, were used to summarize demographic characteristics and to describe the prevalence and patterns of suicidal thoughts and behaviors. While suicidal ideation and attempts represent distinct constructs, further stratification was limited by small cell sizes for specific attempt categories; therefore, analyses focused on a composite suicidality outcome to ensure stable estimation. Methods of suicide attempts were compared descriptively across the three study sites. A modified Poisson model was used to estimate factors associated with suicidality, and suicidality was defined as any suicidal thought or any suicide attempt report. Consistent with prior epidemiologic studies of suicidality, correlates were assessed at the time of study enrollment to characterize social and psychosocial conditions present among individuals with a history of suicidal thoughts or behaviors. The modified Poisson model, which is useful for estimating relative risk by combining a log Poisson regression model with robust variance estimation [61], was fit to the data as opposed to a logistic regression model. A logistic regression model would have been appropriate if suicidality was rare (less than 10%) in these data, but it tends to over-estimate the risk otherwise, rendering the Poisson regression model better in this context [62,63,64]. Analyses focused on estimation of prevalence ratios and their 95% confidence intervals rather than formal hypothesis testing. Bivariate analyses were conducted and variables with a *p*-value greater than 0.25 were considered in the multivariable modeling. Multicollinearity was tested using Variance Inflation Factors (VIFs) at a cut-off point of 10. In case of model comparison, Akaike’s and Bayesian information criteria were used. Stata 15.0 (StataCorp, College Station, TX, USA) was used for all analyses and the statistical significance was set a priori at *p* < 0.05.

## 3. Results

### 3.1. Demographic Characteristics of Study Participants

Of the 300 women, most had attained at least some secondary education (66.0%) and had biological children (62.0%). Of the 62.0% that had children, 81.7% lived with their children. Most participants reported that both of their parents were alive (65.0%), while 36.7% did not live with their parents. A substantially higher proportion of women with primary or lower education compared to those with some secondary or higher education reported either suicidal thoughts or attempts (Table 1).

### 3.2. Prevalence and Patterns of Suicidal Thoughts and Behaviors

The prevalence of lifetime suicidal thoughts was 46.0%, and the prevalence of lifetime suicide attempts was 17.3%. Of the 300 women, 53.3% reported neither suicidal thoughts nor attempts, 29.3% reported suicidal thoughts only, less than 1% reported suicidal attempts only, and 16.7% reported both suicidal thoughts and attempts. Overall, 17.3% reported a lifetime suicide attempt, of which 65.4% were classified as actual attempts, irrespective of whether interrupted or aborted attempts were also reported. Interrupted and aborted attempts each accounted for 23.1% of all reported attempts (Table 2).

### 3.3. Suicide Methods

**Figure 1:**
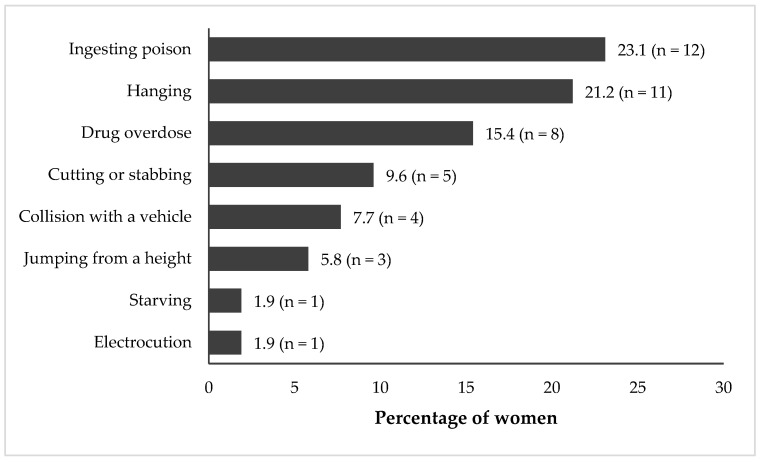
Distribution of suicide attempt methods (proportions based on overall responses, *n* = 52; valid responses, *n* = 45).

The most commonly reported methods of suicide attempts were poison ingestion (23.1%) and hanging (21.2%), while the least commonly reported methods were starving oneself and electrocution (1.9% each) (Figure 1). No differences in the suicidal methods were observed across the three study sites.

### 3.4. Multivariable Analysis of Correlates of Suicidal Thoughts and Behaviors

Self-reported depression, intimate partner violence, and having a consistent partner were associated with suicidal thoughts or behaviors (Table 3).

Adjusting for other model variables, women who self-reported depression were 86% more likely to report suicidal thoughts or attempts (prevalence ratio, PR = 1.86; 95% confidence interval, CI, 1.40–2.48) compared to those who did not report depression, and those who experienced any form of intimate partner violence were 44% more likely to report either suicidal thoughts or attempts (PR = 1.44; 95% CI, 1.04–1.99) compared to women who did not experience any form of violence, whereas women who had consistent sexual partners were less likely to report suicidal thoughts or attempts (PR = 0.78; 95% CI, 0.61–1.01) compared to those who had no partners.

## 4. Discussion

We present a community-based study examining the prevalence of suicidal thoughts and attempts among young women living in poverty in the slums of Kampala, Uganda, using the C-SSRS [37]. The lifetime and past-month prevalence of suicidality were high, at 46.7% and 16.3%, respectively, reflecting substantial unmet mental health need in this population of young women. For context, lifetime measures of suicidality are widely used in epidemiologic research and are consistently identified as among the strongest indicators of vulnerability to future suicidal behavior, even when the timing of onset cannot be precisely established. As such, it is important to recognize that the overall prevalence was higher in our population of young women in Uganda than the findings from high-income countries or even what has been noted in Kenya recently [23,58,59]. This discrepancy may reflect the high population of young women living in poverty and the economic instability they likely face, as well as the limited availability and access to essential resources such as healthcare, education, and employment opportunities [42,46]. These conditions can lead to elevated levels of stress and feelings of depression and hopelessness, and may contribute to elevated vulnerability to suicidal behaviors [50,51].

Across adjusted analyses, we found that self-reported depressive symptoms emerged as the strongest correlate of lifetime suicidality in this sample. Young women who screened positive for depressive symptoms had a substantially higher prevalence of suicidal thoughts or attempts compared with those who did not report depressive symptoms. This finding is consistent with a large body of literature identifying depression as a central correlate of suicidality across diverse settings, including low- and middle-income countries [2,4,5,10], and underscores the importance of mental health screening and treatment within underserved urban communities. However, the availability of mental health services and resources remains very limited in this region, and pervasive stigma surrounding mental illness may further deter individuals from seeking help, even when services are available [21,25].

Experiencing IPV was also independently associated with lifetime suicidality, highlighting the intersection between gender-based violence and mental health vulnerability among young women living in urban poverty. This finding aligns with prior evidence linking IPV and other forms of violence to psychological distress and suicidal thoughts and behaviors in similar settings [2,10]. Having a current sexual partner was associated with lower prevalence of lifetime suicidality, suggesting that, in contexts of poverty and social instability, the presence of a partner may reflect forms of relational and social stability, or even financial stability, that are relevant for mental health, although this association should be interpreted cautiously. Additionally, although lower educational attainment was associated with suicidality in unadjusted analyses, this association attenuated after accounting for psychosocial factors, suggesting that education may operate through broader contextual or mental health pathways. Other factors commonly examined in studies of suicidality [2,4,5,10], including alcohol use, engagement in sex work, and household wealth, were not independently associated with lifetime suicidality in adjusted analyses in this study.

While the prevalence of suicidal ideation in our sample was substantially higher than estimates reported in many other settings, interpretation of these findings must be situated within Uganda’s legal, sociocultural and data-system context. Compared with many countries, Uganda lacks strong vital statistics surveillance programs, which could provide valuable information about the number of completed or attempted suicides. Although retrospective medical record reviews have demonstrated that hospital data can serve as a viable source for estimating fatal and non-fatal suicide attempts [35,65], these approaches do not capture individuals who attempt suicide but do not seek formal medical care. Underreporting is further compounded by persistent mental health stigma, which may discourage disclosure of suicidal thoughts and behaviors. Suicide and attempted suicide have historically been criminalized in Uganda, a legal context that may contribute to stigma, fear of disclosure, and barriers to help-seeking, even where enforcement is inconsistent [66]. Although the prior literature suggests that these laws are rarely enacted, their existence may nonetheless reinforce downstream discrimination and exacerbate stigma surrounding mental health conditions [67]. Uganda also lacks a comprehensive, standalone national suicide prevention strategy, with prevention efforts largely embedded within broader mental health frameworks [41,68]. Religion plays a central role in social life and may simultaneously offer protective social support while reinforcing moral stigma around suicide, potentially shaping disclosure, reporting, and care-seeking behaviors [69,70,71]. Interpretation of national suicide burden is further complicated by weak civil registration and vital statistics systems, meaning available estimates rely largely on modeled or facility-based data and likely underestimate true prevalence, particularly in marginalized urban communities [35,42,72]. Together, these factors suggest that observed prevalence estimates, particularly among young women living in urban slums, may represent underestimates and situated within broader structural constraints rather than as precise reflections of population-level risk. This study is among the first to report on suicide attempt methods reported by young women living in urban slum settings in Uganda. Understanding the specific suicide methods reported by young women in this population can inform contextually relevant prevention strategies [43]. In our study, the three most common methods used were ingesting poison, hanging, and drug overdose. These findings were consistent with those reported by youth in Kenya which found the same reported choices in suicide methods [23]. Additionally, environmental factors related to the availability of certain methods could be modified to reduce the incidence of suicide attempts, particularly ingestion of poisons. Restriction of lethal means may be relevant for women living in slum communities and may directly reduce fatalities from suicide. While lockboxes for organophosphates, as an example of a toxic substance used in suicides, have not been shown to prevent suicide deaths [68], policy regulations on highly toxic organophosphates have shown population-level reduction for suicide fatalities in Asia [43]. Finally, directly addressing mental healthcare access by improving transportation, reducing waitlists and increasing the workforce, are ambitious but achievable goals to reduce premature deaths associated with suicide in low- and middle-income countries [65].

Our findings need to be interpreted with several important limitations in mind. First, the data from the TOPOWA baseline assessment was cross-sectional, precluding causal inference. Second, the use of a self-report survey introduces the possibility of social desirability bias, due to stigma and the pressure of providing socially acceptable responses. Nevertheless, examining correlates measured at the time of assessment among individuals with a lifetime history of suicidality is a common epidemiologic approach for characterizing current social and psychosocial conditions among vulnerable populations. The survey questions were read by a trained research assistant for the participants in a private setting to make the participant as comfortable as possible. Moreover, the sample size was relatively modest. Findings may not be generalizable beyond young women living in similar low-resource urban settings. Additionally, the modest sample size may have contributed to unstable estimates of prevalence as we provided specific analyses of suicidality. Finally, the study’s inclusion and exclusion criteria may have inadvertently excluded women with severe mental health concerns. More specifically, those who presented with self-reported pregnancy, significant intellectual disability, or severe mental illness or substance use requiring hospitalization were excluded from recruitment into the cohort study. Consequently, our findings could underestimate the true prevalence of suicidality within this population. Also for context, although suicidal ideation and suicide attempts are conceptually distinct phenomena, further stratified analyses were limited by sample size; future longitudinal follow-up of this cohort will allow examination of transitions across suicidal phenotypes.

## 5. Conclusions

The TOPOWA study highlights the importance of vigilance regarding any suicidal thoughts and, particularly, suicidal plans among these young women. Since not all suicide attempts are associated with preceding thoughts or plans, it is crucial to consider a broader range of risk factors and warning signs [46]. Our study also identifies factors that were associated with lifetime suicidality, including lower levels of education and parental loss. Having strong social support systems and educational factors should be evaluated for intervention targets for suicide prevention [50].

In the context of Uganda, these findings highlight the critical need for targeted mental health interventions and support systems in communities where the level of suicidality is high. Given the high prevalence of psychological distress and the challenging conditions many young women face, comprehensive strategies that include education and skill training, mental health services, and psychosocial support will be essential [50,73]. These are components of the TOPOWA study that will be examined as soon as data are available. By addressing these factors, we can develop more effective, culturally appropriate interventions that offer sustainable solutions to reduce suicidality in vulnerable populations.

## Figures and Tables

**Table 1:** Baseline demographic characteristics of participants in the TOPOWA cohort study (*N* = 300).

Characteristic	Category	Overall Sample n (%) ^a^	Reported Suicidality n (%) ^b^	*p*
Education level	Primary or lower	102 (34.0)	56 (54.9)	0.040
	Some secondary or higher	198 (66.0)	84 (42.4)	
Age (years)	18–20	144 (48.0)	69 (47.9)	0.677
	21–24	156 (52.0)	71 (45.5)	
Has biological children	No	114 (38.0)	48 (42.1)	0.215
	Yes	186 (62.0)	92 (49.5)	
Household size	≤4	169 (56.3)	74 (43.8)	0.256
	>4	131 (43.7)	66 (50.4)	
Parents’ survival status	Both parents alive	195 (65.0)	93 (47.7)	0.088
	One parent alive	91 (30.3)	37 (40.7)	
	No parents alive	14 (4.7)	10 (71.4)	
Lives with parents	No	176 (58.7)	74 (42.0)	0.056
	Yes	110 (36.7)	56 (50.9)	
	Has no parents	14 (4.6)	10 (71.4)	
Lives with her children	No	34 (11.3)	18 (52.9)	0.419
	Yes	152 (50.7)	74 (48.7)	
	Has no children	114 (38.0)	48 (42.1)	
Generations in household	One	45 (15.0)	17 (37.8)	0.303
	Two	154 (51.3)	71 (46.1)	
	Three or more	101 (33.7)	52 (51.5)	

^a^ Column percentage; ^b^ row percentage; *p*, chi-square test *p*-value.

**Table 2:** Lifetime suicidal prevalence among the TOPOWA cohort participants (*N* = 300).

Suicidal Thoughts and Behavior	*n* (%)
Prevalence of Suicidal Thoughts and Attempts	
Suicidal thought (regardless of attempt)	138 (46.0)
Suicide attempt (regardless of thought)	52 (17.3)
Status of Suicidal Thoughts and Behaviors	
No suicidality (no thought + no attempt)	160 (53.3)
Suicidal thought only (thought + no attempt)	88 (29.3)
Suicide attempt only (no thought + attempt)	2 (0.7)
Suicidal thought + attempt	50 (16.7)
Suicide Attempt	
No attempt	248 (82.7)
Any attempt	52 (17.3)
Actual attempt (regardless of interruption)	34 (11.3)
Interrupted attempt only	12 (4.0)
Aborted (self-interrupted) attempt only	12 (4.0)

**Table 3:** Associations between demographic characteristics and suicidality among the TOPOWA cohort participants (*N* = 300).

Characteristic	Bivariate Analysis		Multivariable Analysis	
	CPR (95% CI)	*p*	APR (95% CI)	*p*
Education level				
Primary or lower	Ref.		Ref.	
Some secondary or higher	0.77 (0.61, 0.98)	0.035	0.84 (0.67, 1.06)	0.138
Has biological children				
No	Ref.		Ref.	
Yes	1.17 (0.91, 1.52)	0.225	0.94 (0.71, 1.23)	0.629
Generations in household				
One	Ref.		Ref.	
Two or more	1.28 (0.86, 1.90)	0.227	1.33 (0.90, 1.95)	0.153
Parents’ survival status				
None	Ref.			
At least one alive	0.64 (0.45, 0.91)	0.013		
Age (years)				
18–20	Ref.			
21–24	0.95 (0.75, 1.21)	0.677		
Household size				
≤4	Ref.			
>4	1.15 (0.90, 1.46)	0.255		
Lives with parents				
No	Ref.			
Yes	1.15 (0.90, 1.47)	0.257		
Lives with her children				
No	Ref.			
Yes	1.09 (0.86, 1.39)	0.479		
Depression				
No	Ref.			
Yes	1.97 (1.47, 2.64)	<0.001	1.86 (1.40, 2.48)	<0.001
Intimate partner violence				
No	Ref.			
Yes	1.42 (1.05, 1.94)	0.024	1.44 (1.04, 1.99)	0.026
Has a current sexual partner				
No	Ref.			
Yes	0.86 (0.67, 1.10)	0.225	0.78 (0.61, 1.01)	0.047
Tested for HIV				
No	Ref.			
Yes	1.77 (0.97, 3.23)	0.065	1.40 (0.72, 2.70)	0.321
Past-month alcohol use				
No	Ref.			
Yes	1.23 (0.94, 1.59)	0.127	1.16 (0.90, 1.48)	0.247
Engaged in sex work				
No	Ref.			
Yes	1.18 (0.68, 2.05)	0.566		
Wealth quintile				
Low	Ref.			
High	1.11 (0.87, 1.42)	0.387		
Quality of life	0.98 (0.96, 0.99)	0.001		

CPR, crude prevalence ratio. APR, adjusted prevalence ratio. CI, confidence interval. *p*, *p*-value. Ref., reference category. Note: Quality of life was excluded from the final multivariable analysis model due to high multicollinearity. In addition, a model that excluded it performed better based on Akaike’s and Bayesian information criteria.

## Data Availability

The data presented in this study are available on reasonable request from the corresponding author and will also be available following the completion of the cohort study.

## Abbreviations

The following abbreviations are used in this manuscript:

CI	Confidence interval
COVID-19	Coronavirus Disease 2019
C-SSRS	Columbia Suicide Severity Rating Scale
HIV/AIDS	Human Immunodeficiency Virus/Acquired Immune Deficiency Syndrome
PR	Prevalence ratio
TOPOWA	The Onward Project on Wellbeing and Adversity
UNCST	Uganda National Council for Science and Technology
UYDEL	Uganda Youth Development Link
